# A View from the Other Side

**DOI:** 10.1007/s44402-026-00034-2

**Published:** 2026-02-05

**Authors:** Mark Rosenfield

**Affiliations:** Ophthalmic and Physiological Optics, https://link.springer.com/journal/44402

As the editor of OPO, I would rarely accept a manuscript based on a single subject, and similarly, the journal seldom, if ever, publishes case reports. But my experiences over the past 3 months have been, to say the least, enlightening, and it is always interesting for a researcher to spend time sitting as an experimental participant, or for a clinician to be the patient for once.

Over the past summer, I was prescribed three doses of oral steroids for a nerve impingement in my back, which did absolutely nothing for my leg pain, but caused the development of cataracts, particularly in my left eye. It also ruled out any possibility of my being picked for the Olympic team! The textbooks often describe having a cataract as looking through a cloudy or foggy window, and that is about right. I want to clean my glasses all the time, even though I just cleaned them. Interestingly, the degree of visual impairment is very variable, depending on the level of illumination and time of day. Patients with cataracts have often complained to me about driving at nighttime, but I found that the effects of light scatter reducing retinal contrast were far worse in the mid-morning and around dusk (in the early Winter of the northern hemisphere), when the low sun had a much greater impact on my vision than the degree of impairment in darkness, or even in the middle of the day.

The negative effect of glare on vision has been well documented, and serves to demonstrate just how poor standard clinical measurements are for evaluating real-world visual resolution. For example, in my left eye, corrected visual acuity (VA) using my habitual spectacles under standard clinical conditions, with good illumination and viewing a high contrast letter chart was 0.10 logMAR (6/7.5 or 20/25 for my American friends who haven’t yet discovered the metric system). Many clinicians would likely regard that as a pretty good level of VA with nothing much to worry about. In fact, many might tell me to wait until the cataract “became ripe” if that was the only measure of vision being recorded. Reducing the luminance by 50% with the addition of a 0.3 log unit neutral density filter before the eye lowered VA to 0.16 logMAR (approximately 6/9). But add two glare sources, using two LED lamps producing a total of approximately 1750 lux, positioned 30° either side of the visual axis, and my VA plummeted to 1.06 logMAR (6/69) and 1.08 logMAR (6/72 or 20/240) with and without the neutral density filter, respectively. And then, just to rub salt into the wound, when switching from a near 100% contrast chart to a 10% contrast chart, with the additional glare, my VA was now worse than 1.10 logMAR (6/72 or 20/240). At this point, I can hear my students say, why didn’t you move closer to the chart to get a proper measure of VA, and they would be quite correct, apart from the fact the glare sources were fixed in place.

The take home message here is that as clinicians, we are doing a very poor job of assessing how our patients see in everyday life. If high contrast VA was the only assessment of vision taken, then in the case of my left eye, everything would seem fine (VA = 6/7.5). But lower the contrast of the object of regard and add additional glare, and then the best corrected VA falls below the threshold for legal blindness. It is also far below the standard required to drive a motor vehicle in any jurisdiction I am aware of. Given this highly variable degree of resolution (thankfully in only one eye), should I be allowed to drive or not? Anyone who happens to live in or near the great state of New Jersey will be relieved to know that I did indeed avoid driving at those times perceived to be most debilitating.

So the take home message is that as eye care practitioners, there is a clear need to reevaluate how we measure visual resolution in the clinical setting. The limitations of the Snellen chart have been well documented [1–4], and yet this 19^th^ century system continues to be used widely. It is hard to explain why optometrists and ophthalmologists are apparently so unwilling to give up this highly flawed method of quantification, given that VA is the most commonly obtained clinical measure of vision. Further, testing with only high contrast optotypes under optimal viewing conditions is not representative of real world demands, such as driving at dusk or identifying small changes in luminance (such as walking down a poorly lit staircase or reading low contrast text from an old and well-worn book). Indeed, nighttime conditions have recently become even more demanding, with the introduction of high intensity LED car headlights. Fortunately, the College of Optometrists has been involved in an evaluation of these lamps [5].

The good news is that by the time you read this editorial, I should have undergone surgical removal of the cataract, and hopefully normal vision will have been restored. In the meantime, I am still struggling (much to my wife’s amusement) to instill drops into my own eye (but I am now confident that my cheek is not infected or inflamed). But just going through the pre-operative procedures has been enlightening. These included the ophthalmological practice trying to convince me to undergo a more complex surgical technique that lacks research support to justify its usage, and then telling me that my post-operative near vision would be significantly worse if I didn’t receive these advanced procedures (which coincidently were not covered by insurance). But even relatively simple things that were omitted served as excellent reminders for me, such as failing to tell a patient with a cataract not to drive home after having their pupils dilated, (don’t worry – I didn’t) or even giving me any indication of how long the dilation was likely to last. Additionally, they “forgot” to tell a friend of mine who underwent cataract removal a few weeks ago and now needs a near-vision correction for the first time, to be extra cautious when navigating stairs or the edge of the pavement, but instead let him discover this all by himself after falling on the steps.

As they say, all experience is good experience, and the next time I encounter a patient or an experimental participant with a cataract, hopefully I will be more sympathetic and have a much better understanding of what they are experiencing. Perhaps every few years, we should all have a rigid contract lens or a gonioscopy lens placed on our eye, or undergo a central AND peripheral visual field assessment, just to remind ourselves what we put our patients through. Similarly, try sitting as a subject for an experiment once in a while, to see just how “minimal” the level of discomfort or risk really is, to find out whether you can sit still and perform the task adequately for the required duration and precisely how it all feels. Indeed, that is probably good advice for all medical specialties, and I will leave it to your imagination and degree of sadistic intent, as you consider what non-ophthalmic clinical procedures need to be inflicted upon the medical professionals who perform them on a regular basis.

## Data Availability

No datasets were generated or analysed during the current study.
